# Halogen as Template to Modulate the Structures of the Nanocage-Based Silver(I)-Thiolate Coordination Polymers

**DOI:** 10.3390/molecules31020331

**Published:** 2026-01-19

**Authors:** Chunhong Tan, Li Tang, Jiajia Tan, Jinrong Zhang, Juan Zhou, Linmao Yin, Xiao-Feng Wang

**Affiliations:** 1 School of Chemistry and Chemical Engineering, University of South China, Hengyang 421001, China; 2015002077@usc.edu.cn (C.T.);; 2 School of Resources Environment and Safety Engineering, University of South China, Hengyang 421001, China; 3 School of Mechanical Engineering, University of South China, Hengyang 421001, China; 4 School of Civil Engineering, University of South China, Hengyang 421001, China; 2017000005@usc.edu.cn

**Keywords:** halogen template, multi-sliver cages, coordination polymers, crystal structure

## Abstract

By the reaction of AgNO_3_, 2-methyl-2-propanethiol (HS*t*Bu), with various-sized halogen ions as templates, three multi-nuclear silver-thiolate cluster-based chain-like coordination polymers, [Ag_6_(*μ*-SBu)_6_]_n_ (**USC-CP-2**), [Ag_6_(*μ*-S*t*Bu)_5_Br]_n_ (**USC-CP-4**) and [Ag_14_(*μ*-S*t*Bu)_12_I_2_]_n_ (**USC-CP-3**) constructed by different Ag(I)-nanocages, have been synthesized and characterized by X-ray diffraction analyses. With F^−^, Cl^−^ or without template, **USC-CP-2** exhibits a one-dimensional structure composed of detached Ag_6_-cages, absent of fluoride or chloridion. While with Br^−^ and I^−^, **USC-CP-4** and **USC-CP-3**, two distinct halogen-templating multi-sliver cages-based chain-like polymeric structures have been observed, which are a mono-Br^−^ encapsulated Ag_8_-cage, or a dual-I^−^ embedded Ag_16_-cage, respectively. In these three compounds, the multi-Ag(I) cages were self-assembled by Ag-S bonds through bridged *μ*_2_-S*t*Bu ligands, and stabilized argentophilic interactions between neighboring silver atoms. This study demonstrates that the halide anions of varying sizes play a critical role in inducing the nucleation and structural evolution of the silver-thiolate clusters.

## 1. Introduction

Silver(I)-thiolate clusters coordination complexes, as an important class of organometallic compounds, have garnered significant research interest due to their diverse structural architectures [1]. Crucially, in contrast to conventional nanoparticles, atomically precise Ag(I)-thiolate clusters can be used as well-defined building blocks to construct coordination compounds with tailored functionalities for promising applications, such as multifunctional biomaterials, semiconductor devices, and luminescent materials [2,3,4,5,6,7,8]. The hierarchical structural patterns are constructed through the self-assembly of Ag(I) nodes and thiolate-based organic linkers, ranging from the discrete cluster to high-dimensional structures, including 1-D chains, 2-D sheets, and 3-D frameworks [3,4,5,6,9]. The structural diversity of Ag(I)-thiolate coordination complexes was collectively dictated by many factors, beyond conventional metal-ligand coordination, also including the synergistic π-π stacking, argentophilic Ag⋯Ag interactions, and dynamic anion bridging. The influencing factors are detailed as follows: (i) Ag(I)-to-ligand ratios, (ii) pH values to protonate/deprotonate thiolates, (iii) steric bulk of ancillary side substituent groups, (iv) solvent polarity and Lewis basicity, and (v) nature of counter-anions (hard vs. soft, mono- vs. poly-dentate) [4,5,6]. Thus, their self-assembly often becomes complex and is associated with uncontrolled processes, making for rational design and targeted functionalization difficult and challenging.

To address this limitation, the introduction of templating anion has emerged as a powerful strategy for directing the formation of high-dimensional Ag(I)-thiolate coordination structures [10,11,12,13,14]. The use of anion templates offers several advantages: (i) precise control of cluster nuclearity and geometry, (ii) enhancing structural stability through its charge balance to cationic metal centers, and (iii) incorporating unique physical properties of functional anions. To date, a wide range of anionic templates have been employed, ranging from simple spherical (S^2−^ and halides X^−^ = F^−^, Cl^−^, Br^−^ and I^−^), planar trigonal (NO_3_^−^ and CO_3_^2−^), to tetrahedral species moieties (PS_4_^3−^ and VO_4_^3−^) [15,16,17,18,19].

In our previous research, we reported on the incorporation of iodide to induce the formation of the Ag_16_-cage, which resulted in the corresponding chain-type Ag(I)-thiolate coordination polymer [16]. Herein, this work systematically investigated the inducing template effects of halide anions on the structural control of self-assembly of chain-like Ag(I)-thiolate coordination polymers. Through employing variation in the halide template (F^−^, Cl^−^, Br^−^ and I^−^), three distinct structures were obtained: [Ag_6_(*μ*-S*t*Bu)_6_]_n_ (**USC-CP-2**), [Ag_6_(*μ*-S*t*Bu)_5_Br]_n_ (**USC-CP-4**) and [Ag_14_(*μ*-S*t*Bu)_12_I_2_]_n_ (**USC-CP-3,** where USC-CP stands for University of South China coordination polymer). Without or with smaller F^−^/Cl^−^ ions as template, the formation of **USC-CP-2** has been obtained, which is a one-dimensional bead-like polymer composed of the detached Ag_6-_cages. While larger Br^−^/I^−^ ions were introduced, **USC-CP-4** and **USC-CP-3** exhibit one-dimensional structures based on edge-shared Ag_8-_ or Ag_16_-cages that encapsulate mono-Br^−^ or dual-I^−^ anions, respectively. The structural comparison indicated that halides have a significant impact on the structures of multi-silver cages. More specifically, using halide templates of different sizes resulted in a progressive increase in the nucleation of silver clusters formed, from six to sixteen, thereby altering the corresponding chain structures. This study provides fundamental insights into the anion-directed assembly of Ag(I)-thiolate coordination polymers and the potential to guide the establishment of structure-property relationships for the future design of the cognate functional materials.

## 2. Experimental Section

### 2.1. Materials and Methods

All reagents and solvents used were received from commercial suppliers without further purification.

The elemental analyses (C, H, and N) were performed with a Vario Micro CHNOS Elemental Analyzer (manufactured by Elementar Analysensysteme GmbH, Langenselbold, Hessen, Germany). The powder X-ray diffraction (PXRD) data were collected on a DMAX-2500 diffractometer with Cu Kα (λ = 1.5418 Å) (produced by Rigaku Corporation, Tokyo, Japan). The thermogravimetric analysis was performed on NETZSCH TG 209F3 (manufactured by NETZSCH-Gerätebau GmbH, Selb, Bavaria, Germany) in an N_2_ atmosphere.

### 2.2. Synthetic Procedures

#### 2.2.1. Preparation of [Ag_6_(*μ*-S*t*Bu)_6_]_n_ (**USC-CP-2**)

***F^−^ ion as template.*** Sodium ethylate (0.028 g, 0.4 mmol) and 2-methyl-2-propanethiol (0.043 mL, 0.4 mmol) were dissolved in 6 mL of ethanol. After stirring for half an hour, AgNO_3_ (0.034 g, 0.2 mmol) dissolved in 1 mL water and 2 mL ethanol was added in slowly to the solution. The solution immediately turned milky white and more flocs were formed. Then add 2 mL of acetone and 1 mL ethanol solution of NaF (0.008 g, 0.2 mmol). The turbid liquid was sealed in a 20 mL Teflon-lined autoclave and heated at 130 °C for 33 h. After the autoclave was cooled to room temperature, strip-shaped colorless crystals of **USC-CP-2** were separated, with a yield of 51% (based on Ag). Anal. Calcd for Ag_6_S_6_C_24_H_54_: C 24.38, H 4.60; found: C 24.32, H 4.72%.

***Cl^−^ ion as template.*** The same synthesis procedure was used, replacing NaF with NaCl (0.012 g, 0.2 mmol). Yield: 50%

***No template.*** A similar synthesis procedure was used, without adding a template agent. Yield: 51%.

#### 2.2.2. Preparation of [Ag_6_(*μ*-S*t*Bu)_5_Br]_n_ (**USC-CP-4**)

Sodium ethylate (0.028 g, 0.4 mmol) and 2-methyl-2-propanethiol (0.043 mL, 0.4 mmol) were dissolved in 6 mL of ethanol. After stirring for half an hour, AgNO_3_ (0.034 g, 0.2 mmol) dissolved in 1 mL water and 2 mL ethanol was added in slowly. The solution immediately turned milky white and more flocs were formed. Then add 2 mL of acetone and 1 mL ethanol solution of tetraphenylphosphine bromide (0.025 g, 0.06 mmol). The turbid liquid was sealed in a 20 mL Teflon-lined autoclave and heated at 130 °C for 33 h. After the autoclave was cooled to room temperature, massive colorless crystals of **USC-CP-4** were separated with a yield of 10% (based on Ag). Anal. Calcd for Ag_6_S_5_C_20_H_45_Br: C 20.48, H 3.87; found: C 20.65, H 3.65%.

#### 2.2.3. Preparation of [Ag_14_(*μ*-S*t*Bu)_12_I_2_]_n_ (**USC-CP-3**)

Sodium ethylate (0.028 g, 0.4 mmol) and 2-methyl-2-propanethiol (0.043 mL, 0.4 mmol) were dissolved in 6 mL of ethanol. After stirring for half an hour, AgNO_3_ (0.034 g, 0.2 mmol) dissolved in 1 mL water and 2 mL ethanol was added in slowly. The solution immediately turned milky white and more flocs were formed. Then add 2 mL acetone and 1 mL ethanol solution of potassium iodide (0.033 g, 0.2 mmol). The turbid liquid was sealed in a 20 mL Teflon-lined autoclave and heated at 130 °C for 33 h. After the autoclave was cooled to room temperature, massive light yellow crystals of 3 were separated yield: 19% (based on Ag). Anal. Calcd for Ag_14_S_12_C_48_H_108_I_2_: C 20.34, H 3.84; found: C 20.22, H 3.93%.

### 2.3. X-Ray Crystallographic Analysis

All data were collected on a Rigaku SCXmini CCD diffractometer equipped with graphite-monochromated Mo-Kα radiation source (produced by Rigaku Corporation, Tokyo, Japan) (λ = 0.71073 Å) by ω scan mode. The structures were solved by the direct method using the SHELXTL Version 5 package of crystallographic software, and refined with a full-matrix least-squares refinement on F^2^. Metal atoms were located from the E-maps and refined anisotropically. The other non-hydrogen atoms were located by the difference Fourier maps based on these atomic positions and refined anisotropically. Hydrogen atoms were added according to the theoretical models. The crystallographic data have been deposited into the Cambridge Crystallographic Data Centre, CCDC no. 2435678, for **USC-CP-4**. Copies of this information may be obtained free of charge from the Director, 12 Union Road, Cambridge CB2 1EZ, UK; fax: +44-1223-336033; e-mail: deposit@ccdc.cam.ac.uk. Crystal data collection and refinement parameters are summarized in Table 1, and their selected bond lengths and angles are provided in Tables S1–S3, respectively.

## 3. Results and Discussion

### 3.1. Synthesis

Polynuclear silver(I)-thiolate clusters are of special interest not only due to the central role they play in multifunctional biomaterials but also due to interest in the establishment of luminescent structural correlations. A variety of multi-silver(I) complexes with different clusters have been reported. When KI was added into the reaction system of AgNO_3_ and HS*t*Bu, the dual-I^−^ embedded Ag_16_-cage based structure was isolated in our previous work [16], in which the strong interaction of Ag⋯I (the shortest distance of 2.99 Å) was observed. This effect should also be exhibited by other halide and silver ions. Thus, we attempted to systematically investigate the impact of other halogens (F^−^, Cl^−^ and Br^−^) on the structure of silver(I)-thiolate clusters. However, when the solvent polarity, temperature and reactant ratio of the reactants in reaction systems with F^−^ or Cl^−^ as templates were extensively regulated, Ag_6_-cage based **USC-CP-2** was isolated as the sole pure-phase product. Interesting, it could also be obtained without the addition of templates. In **USC-CP-2**, neither the F^−^ nor Cl^−^ ions are encapsulated within the multi-silver cages. In contrast, analogous reactions with Br^−^ or I^−^ anions yielded **USC-CP-4** and **USC-CP-3**, which are based on Ag_8_- and Ag_16_-cages that encapsulate mono-Br^−^ and dual-I^−^ anions, respectively. This stark difference can be attributed to the high solvation energy, small ionic radii of F^−^/Cl^−^, as well as the weaker interaction with Ag(I) than that of the Br^−^/I^−^; thus, F^−^/Cl^−^ ultimately did not serve as a template for regulating the putative multi-silver(I) cages [20].

It is important to note that this may be due to the strong binding affinity between silver and thiol groups, coupled with the high stability of compound **USC-CP-2**. It has been observed that the synthesis of **USC-CP-3** and **USC-CP-4** is accompanied by the partial formation of **USC-CP-2**, which consequently reduces the yield of the target products. In addition, despite endeavors to optimize the procedure through adjustments to the sequence and amount of bromide and iodide addition, no substantial enhancement in yield was observed.

### 3.2. Crystal Structure Description

Even though the structures of **USC-CP-2** and **USC-CP-3** have been reported in our previous work [16], it is merited to indicate the detailed dissimilarities induced by the effects of templates.

#### 3.2.1. Molecular Structure of [Ag_6_(*μ*-S*t*Bu)_6_]_n_ (**USC-CP-2**)

**Figure 1:**
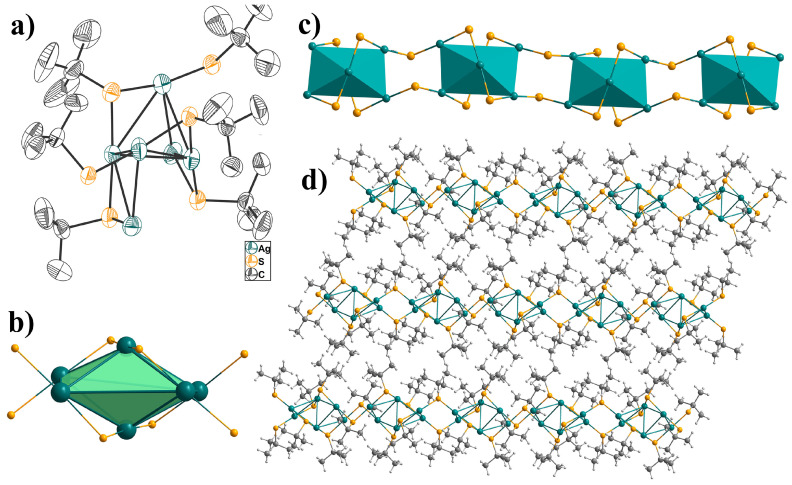
(**a**) ORTEP view of the asymmetric unit in **USC-CP-2** with 50% probability ellipsoids; (**b**) polyhedral hexanuclear silver cage supported by *μ*_2_-S*t*Bu; (**c**) the 1-D beaded-like chain; (**d**) off-set packing of the chains in **USC-CP-2** (Ag turquoise, S golden, C gray, H light gray).

Single-crystal X-ray diffraction analysis indicates that **USC-CP-2** crystallizes in the triclinic *P*$\bar{1}$ space group. The asymmetric unit consists of six independent Ag^+^ atoms and six deprotonated *μ*-S*t*Bu^−^ (Figure 1a). All silver atoms are linearly coordinated by two sulfur atoms from a *μ*_2_-bridging S*t*Bu^−^ ligand. The Ag-S bond distances are in the range of 2.369(2)-2.400(1) Å, with a mean value of 2.385 Å. The distances of Ag⋯Ag range from 3.059(6) to 3.336(7) Å, which are longer than the Ag-Ag distance in metallic silver (2.889 Å) but shorter than the sum of the van der Waals radii (3.44 Å), indicating the presence of significant argentophilic interactions [21]. Thus, it can be considered to form a thiol-bridging Ag_6_-cage. These discretely adjacent Ag_6_-cages are linked by two bridging *μ*_2_-S*t*Bu^−^ into a one-dimensional bead-like coordination chain, and further assembled into a three-dimensional supramolecular structure via weak intermolecular interactions of methyl groups from *μ*-S*t*Bu^−^ ligands [22,23,24,25,26].

#### 3.2.2. Molecular Structure of [Ag_6_(*μ*-S*t*Bu)_5_Br]_n_ (**USC-CP-4**)

**Figure 2:**
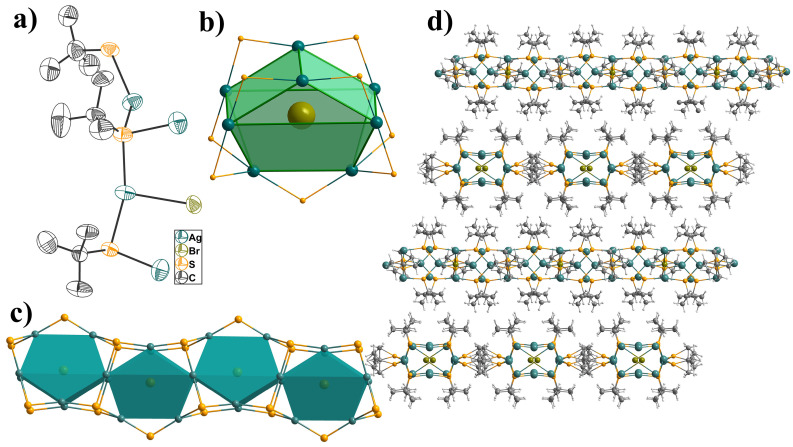
(**a**) ORTEP view of the asymmetric unit in **USC-CP-4** with 50% probability ellipsoids; (**b**) polyhedral octanuclear silver cage supported by *μ*_2_-S*t*Bu and Br^−^ template; (**c**) the 1-D wave-chain; (**d**) interlaced packing of the chains in **USC-CP-4** (Ag turquoise, S golden, Br olive green, C gray, H light gray).

**USC-CP-4** crystallizes in the tetragonal system with the $I\bar{4}2d$ space group. The asymmetric unit comprises three independent silver atoms, two and a half deprotonated StBu^−^ ligands and half a bromide anion (Figure 2a). And as depicted in Figure 2b, it can be expanded as a central bromide anion encapsulating eight Ag atoms formed Ag_8_-cage as secondary building units. Of the eight Ag atoms in the cage display different coordination geometries: four in V-shaped AgS_2_, two in distorted trigonal pyramidal AgS_3_ and two in a nearly triangular AgS_2_Br geometry, respectively. The S*t*Bu^−^ adopted two coordination modes: the *μ*_3_-bridging three Ag(I) atoms and *μ*_2_-bridging two. The Ag-S bond lengths range from 2.371(4) to 2.589(8) Å, with an average value of 2.436 Å. The Br^−^ anion acts as a V-shaped configuration to bridge two Ag^+^ ions with an Ag-Br distance of 2.871(2) Å, which is substantially shorter than the sum of the relevant van der Waals radii (3.57 Å) [27]. Similarly to **USC-CP-2**, there are argentophilic interactions in the Ag_8_-cage, with the Ag-Ag distances ranging from 3.178(3) to 3.220(3) Å. Each Ag_8_-cage connects two neighboring cages through edge-sharing to form a one-dimensional wave chain, and is further formed into a three-dimensional supramolecular structure via weak intermolecular interactions of methyl groups from S*t*Bu^−^ ligands.

#### 3.2.3. Molecular Structure of Ag_14_(*μ*-S*t*Bu)_12_I_2_]_n_ (**USC-CP-3**)

**Figure 3:**
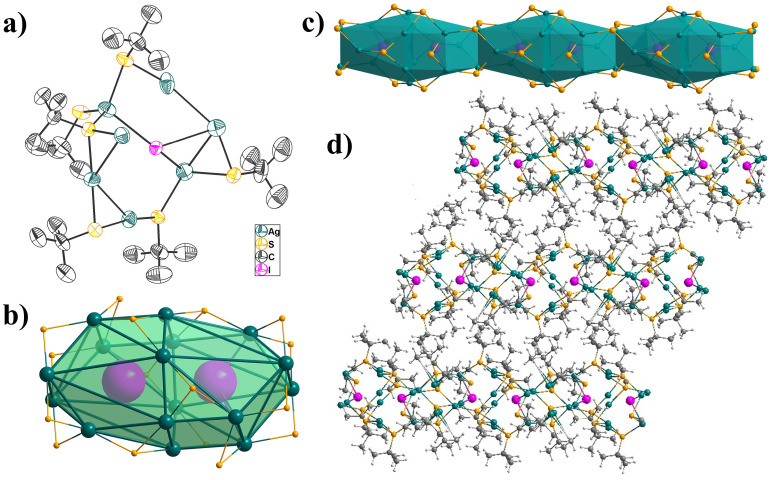
(**a**) ORTEP view of the asymmetric unit in **USC-CP-3** with 50% probability ellipsoids; (**b**) polyhedral sixteen-nuclear silver cage supported by *μ*_2_-S*t*Bu; (**c**) the 1-D linear chain; (**d**) offset packing of the chains in **USC-CP-3** (Ag turquoise, S golden, I purplish red, C gray, H light gray).

The **USC-CP-3** crystallizes in the monoclinic *P*2_1_/c space group. The asymmetric unit contains seven independent Ag(I) atoms, six deprotonated S*t*Bu^−^ ligands, and one iodide (I^−^) anion (Figure 3a), which can be expanded as a dual-iodide enclosed Ag_16_-cage. Similarly to **USC-CP-4**, there are two coordination modes S*t*Bu^−^ in **USC-CP-3**: the *μ*_3_- and *μ*_2_-bridging fashions. The Ag-S bond lengths range from 2.269(6) to 2.695(2) Å, with an average one of 2.444 Å. The I^−^ weakly coordinates with three silver atoms in a trigonal pyramidal fashion. The Ag-I distances are 2.991(2), 3.210(2), and 3.256(1) Å, respectively. The short Ag-Ag distances, ranging from 2.980(7) to 3.344(2) Å, also confirmed the presence of weak metal-metal interactions. Within the Ag_16_-cage, six Ag(I) atoms are coordinated in a distorted tetrahedral AgS_3_I geometry, while the other ten Ag(I) atoms possess the V-shaped AgS_2_ coordination environment. Every Ag_16_-cage is linked to two adjacent cages by edge-sharing to generate a one-dimensional linear chain. The neighboring chains pack together via weak intermolecular forces of methyl groups from S*t*Bu^−^ ligands to form light yellow crystals.

### 3.3. Structural Comparison

All three coordination polymers exhibit nano-cages based on one-dimensional beaded-like chain structures constructed by Ag-S coordination bonds and sustained by argentophilic interactions. However, the introduction of halide anions as structure-directing templates results in significant differences in the supra-molecular building units (SBU) and their overall chain architectures. Using F^−^ or Cl^−^ as the template agent, a neutral one-dimensional chain is constructed from Ag_6_-cage SBUs in the absence of F^−^ and Cl^−^, where all the silver ions adopt an approximately linear coordination geometry. When bromide anions are used as templates, **USC-CP-4** features mono-Br^−^ inside edging-sharing Ag_8_-cage SBUs, where the silver ions display linear, trigonal, and trigonal pyramidal coordination environments. When iodide anions replace bromide, **USC-CP-3** is assembled based on dual-I^−^ inside edging-sharing Ag_16_-cage SBUs, with silver ions exhibiting tetrahedral and V-shaped geometries.

These variations in the bonding configurations and coordination numbers of the central atoms result in distinct Ag-S bond lengths and Ag⋯Ag distances in the three structures. In detail, the Ag-S bond distances range from 2.369(2) to 2.400(1) Å in **USC-CP-2**, 2.371(4) to 2.589(8) Å in **USC-CP-4**, and 2.269(6) to 2.695(2) Å in **USC-CP-3**, respectively. Meanwhile, the Ag⋯Ag separations span 3.059(6)-3.336(7) Å in **USC-CP-2**, 3.178(3)-3.220(3) Å in **USC-CP-4**, and 2.980(7)–3.344(2) Å in **USC-CP-3**, respectively, which are consistent with literature values [28,29,30,31,32,33]. The data indicate that the use of bromide and iodide templates leads to more complex crystal structures and a broader distribution of bond lengths. This outcome may be attributed to the tendency of silver ions, when coordinating with *μ*-S*t*Bu, to encapsulate halide anions within silver cages while directing the bulky tert-butyl groups outward. The larger ionic radii of bromide and iodide can effectively support and stabilize the resulting architectures, whereas the smaller fluoride and chloride ions fail to stabilize larger clusters, leading only to the more compact Ag-S framework observed in **USC-CP-2**.

### 3.4. Stability Studies

**Figure 4:**
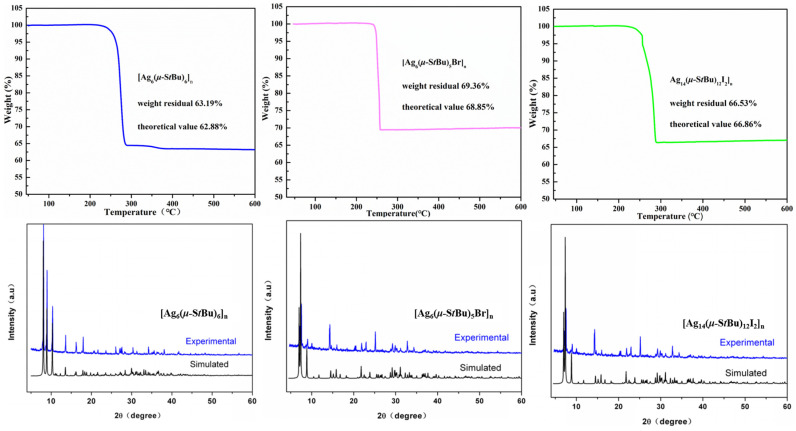
Powder X-ray diffraction patterns (**top**) and thermal gravimetric analyses (**bottom**) of **USC-CP-2**, **USC-CP-4** and **USC-CP-3**.

The crystals of the three CPs mentioned above can remain undamaged after being stored in air for three months or immersed in water for half a month. As depicted in Figure 4, their experimental powder X-ray diffraction patterns perfectly match the simulated one in peak positions. Thus confirm their stability as well as the phase purity of the three CPs. Simultaneously, a comparison of the PXRD pattern of **USC-CP-4** and silver iodide confirmed the absence of AgI impurities in **USC-CP-4** (Figure S4). In addition, thermal gravimetric analysis of coordination polymers **USC-CP-2**, **USC-CP-4** and **USC-CP-3** was performed under a nitrogen atmosphere to evaluate their thermal stability (Figure 4). The thermograms reveal a consistent thermal event initiating around 250 °C, which is attributed to the sublimation of the coordination polymers. Following this initial weight loss, the frameworks undergo progressive decomposition. The pyrolysis process culminates in the formation of stable inorganic residues at 600 °C, with residual masses of 63.19%, 69.36%, and 66.53% for **USC-CP-2**, **USC-CP-4** and **USC-CP-3**, respectively. These residual masses correspond closely to the theoretical values calculated for the formation of silver sulfide (Ag_2_S) from the respective complexes (calcd.: 62.88%, 68.85%, 66.86 wt%), strongly indicating that the final decomposition product is Ag_2_S.

## 4. Conclusions

In summary, three one-dimensional chain-like coordination polymers constructed from different multinuclear Ag(I)-thiolate nanocages have been successfully synthesized via the reactions of AgNO_3_, HS*t*Bu, and halide anions as templates, and characterized by single crystal X-ray diffraction analyses. With smaller halogen (F^−^/Cl^−^) or without template agents, the Ag_6_-cages form a one-dimensional beaded-like chain structure, which is free of fluoride or chloridion. While with larger size Br^−^ as additives, the mono-Br embedded Ag_8_-cages based wave-chain structure is obtained. The largest halogen, I^−^, employed as a template, leads to the formation of the dual-I^−^ encapsulated Ag16-cage-based linear coordination chain. There are argentophilic interactions between neighboring silver atoms, which stabilize these diverse multi-Ag(I) cages. The size of the halide anion is shown to be critical, directly influencing the nucleation process and guiding the structural evolution of the multi-nuclear Ag(I)-thiolate cages and their corresponding chain-like coordination polymers. This study clearly demonstrates that larger halide anions act as effective templates to promote the formation of higher-nuclearity silver-thiolate nano-cages. Future work will extend this methodology to other thiolate ligands, thereby investigating the generality and scope of this templating effect.

## Figures and Tables

**Table 1:** Crystallographic data for **USC-CP-4**.

Parameter	USC-CP-4
Formula	C_20_H_45_Ag_6_S_5_Br
Mr	1172.99
Cryst system	Tetragonal
Space group	$I\bar{4}2d$
*a*/Å	12.208(3)
*b*/Å	12.208(3)
*c*/Å	44.948(2)
*α*/°	90
*β*/°	90
*γ*/°	90
*V*/Å^3^	6699(3)
Z	8
*D*c/g cm^−3^	2.326
*μ*/mm^−1^	4.954
*F*(000)	4496
R(int)	0.0572
Total reflections	28,639
Unique reflections	3917
*I* > 2*σ*(*I*)	3781
*R*_1_	0.0721
*wR*_2_	0.2466
*S*	1.086

## Data Availability

The original contributions presented in this study are included in the Supplementary Material. Further inquiries can be directed to the corresponding authors.

## Supplementary Materials

The supporting information can be downloaded at: https://www.mdpi.com/article/10.3390/molecules31020331/s1. Figures S1–S3: FT-IR spectra of **USC-CP-2, USC-CP-4** and **USC-CP-3**; Figure S4: Powder X-ray diffraction patterns of AgI. Tables S1–S3: Bond lengths (Å) and angles (°) for **USC-CP-2, USC-CP-4** and **USC-CP-3**.

## References

[1] Wang Q., Dong S.L., Tao D.D., Li Z., Jiang Y.B. (2021). Ag(I)-thiolate coordination polymers: Synthesis, structures and applications as emerging sensory ensembles. Coord. Chem. Rev..

[2] Fukuoka M., Takashima Y., Akamatsu K., Demessence A., Tsuruoka T. (2024). Structural transformation of silver(I)–thiolate coordination polymer film at solid–liquid interfaces. CrystEngComm.

[3] Chakraborty I., Pradeep T. (2017). Atomically Precise Clusters of Noble Metals: Emerging Link between Atoms and Nanoparticles. Chem. Rev..

[4] Hawila S., Massuyeau F., Gautier R., Fateeva A., Lebègue S., Kim W.J., Ledoux G., Mesbah A., Demessence A. (2023). Tuning the 1D-2D dimensionality upon ligand exchange in silver thiolate coordination polymers with photoemission switching. J. Mater. Chem. B.

[5] Xu T.Y., Si W.D., Zhang C.K., Han B.L., Wang Z., Tung C.H., Sun D. (2025). Photoswitchable Arylazopyrazole-Functionalized Ag_8_ Nanoclusters with Light-Modulated Photoluminescence. J. Am. Chem. Soc..

[6] Zhang W.F., Ye G.M., Liao D.H., Chen X.L., Lu C.Y., Nezamzadeh-Ejhieh A., Khan M.S., Liu J.Q., Pan Y., Dai Z. (2022). Recent advances of silver-based coordination polymers on antibacterial applications. Molecules.

[7] Kaur N., Tiwari P., Kapoor K.S., Saini A.K., Sharma V., Mobin S.M. (2020). Metal-organic framework based antibiotic release and antimicrobial response: An overview. CrystEngComm.

[8] Medici S., Peana M., Crisponi G., Nurchi V.M., Lachowicz J.I., Remelli M., Zoroddu M.A. (2016). Silver coordination compounds: A new horizon in medicine. Coord. Chem. Rev..

[9] Veselska O., Demessence A. (2018). d^10^ Coinage metal organic chalcogenolates: From oligomers to coordination polymers. Coord. Chem. Rev..

[10] Biswas S., Das S., Negishi Y. (2023). Progress and prospects in the design of functional atomically-precise Ag(I)-thiolate nanoclusters and their assembly approaches. Coord. Chem. Rev..

[11] Dar W.A., Jana A., Sugi K.S., Paramasivam G., Bodiuzzaman M., Khatun E., Som A., Mahendranath A., Chakraborty A., Pradeep T. (2022). Molecular Engineering of Atomically Precise Silver Clusters into 2D and 3D Framework Solids. Chem. Mater..

[12] Das A.K., Biswas S., Manna S.S., Pathak B., Mandal S. (2021). Solvent-Dependent Photophysical Properties of a Semiconducting One-Dimensional Silver Cluster-Assembled Material. Inorg. Chem..

[13] Hu F., Yang G., Zheng L.M., Liang G.J., Wang Q.M. (2025). Deciphering icosahedra structural evolution with atomically precise silver nanoclusters. Science.

[14] Hajda A., Guha R., Copp S.M., Olesiak-Bańska J. (2025). Two-photon brightness of NIR-emitting, atomically precise DNA-stabilized silver nanoclusters. Chem. Sci..

[15] Wei Z.Q., Xiao F.X. (2023). Photosensitization Efficiency Modulation of Atomically Precise Silver Nanoclusters for Photoelectrocatalysis. Inorg. Chem..

[16] Tan C.H., Lu M.H., Zhou T., Fang Z., Zhou J., Wang X.-F., Wang G.Q., Lin Y.W., Rocha J. (2025). Improved pleiotropic antibacterial activity of Ag(I)-thiolate coordination polymers via iodide encapsulation in multinuclear silver nano-cages. Mater. Today Bio..

[17] Horita Y., Ishimi M., Negishi Y. (2023). Anion-templated silver nanoclusters: Precise synthesis and geometric structure. Sci. Technol. Adv. Mater..

[18] Healy C., Schmitt W. (2018). Multicomponent halide templating: The effect of structure-directing agents on the assembly of molecular and extended coordination compounds. Coord. Chem. Rev..

[19] Xie Y.P., Jin J.L., Lu X., Mak T.C.W. (2015). High-nuclearity silver thiolate clusters constructed with phosphonates. Angew. Chem. Int. Ed..

[20] Wang Q.M., Lin Y.M., Liu K.G. (2015). Role of anions associated with the formation and properties of silver clusters. Acc. Chem. Res..

[21] Kumar A.S.K., Tseng W.B., Arputharaj E., Huang P.J., Tseng W.L., Bajda T. (2023). Covalent organic framework nanosheets as an enhancer for light-responsive oxidase-like nanozymes: Multifunctional applications in colorimetric sensing, antibiotic degradation, and antibacterial agents. ACS Sustain. Chem. Eng..

[22] Li S., Du X.S., Li B., Wang J.Y., Li G.P., Gao G.G., Zang S.Q. (2018). Atom-Precise Modification of Silver(I) Thiolate Cluster by Shell Ligand Substitution: A New Approach to Generation of Cluster Functionality and Chirality. J. Am. Chem. Soc..

[23] Tao Y., Luan N., Yang C.Y., Sun J.Y., Li K., Dai X., Zhang H.L., Chai Z.F., Wang S., Wang Y.X. (2024). Incorporation of the ^99^TcO_4^−^_ Anion within the Ag_24_(C≡CtBu)_20_^4+^ Cluster Unveiling the Unique Shell-to-Core Charge Transfer. J. Am. Chem. Soc..

[24] Biswas S., Das A.K., Mandal S. (2023). Surface Engineering of Atomically Precise M(I) Nanoclusters: From Structural Control to Room Temperature Photoluminescence Enhancement. Acc. Chem. Res..

[25] Ma A.L., Du W.J., Wang J.W., Jiang K.F., Zhang C., Sheng W.H., Zheng H.Y., Jin R.C., Wang S.X. (2023). Transforming Silver Nanoclusters from Racemic to Homochiral via Seeded Crystallization. J. Phys. Chem. Lett..

[26] Alhilaly M.J., Huang R.W., Naphade R., Alamer B., Hedhili M.N., Emwas A.H., Maity P., Yin J., Shkurenko A., Mohammed O.F. (2019). Assembly of Atomically Precise Silver Nanoclusters into Nanocluster-Based Frameworks. J. Am. Chem. Soc..

[27] Pan L., Ye S., Huang R.H., Wang M.Y., Lin P.X., Zhang H.R., Wang D.P. (2024). Luminescence Properties and Theoretical Modeling of the Ag_3_ Cluster Confined inside the D6r Cavity of Faujasite Zeolite: Implications for the Design of Tunable Emission Phosphor. ACS Appl. Nano Mater..

[28] Mondal S., Sahoo R., Behera J., Das M.C. (2024). Advances on silver-based MOFs and/or CPs and their composites: Synthesis strategies and applications. Coord. Chem. Rev..

[29] Sunada Y., Yamaguchi K., Suzuki K. (2022). “Template synthesis” of discrete metal clusters with two-or three-dimensional architectures. Coord. Chem. Rev..

[30] Du W.J., Deng S.Y., Chen S., Jin S., Zhen Y.R., Pei Y., Zhu M.Z. (2021). Anisotropic Evolution of Nanoclusters from Ag_40_ to Ag_45_: Halogen- and Defect-Induced Epitaxial Growth in Nanoclusters. J. Phys. Chem. Lett..

[31] Yonesato K., Ito H., Itakura H., Yokogawa D., Kikuchi T., Mizuno N., Yamaguchi K., Suzuki K. (2019). Controlled Assembly Synthesis of Atomically Precise Ultrastable Silver Nanoclusters with Polyoxometalates. J. Am. Chem. Soc..

[32] Luo J., Xu H., Liu Y., Zhao Y., Daemen L.L., Brown C., Wang H., Hu Y.J., Liu D.J. (2014). Hydrated silicotungstate-directed assembly of a 3D silver-thiolate framework with enhanced CO_2_ selectivity. Chem. Commun..

[33] Bonačić-Koutecký V., Mitrić R., Bügel F., Schlauch M., Falk M., Griesbeck A. (2012). Optical properties and dynamics of small silver clusters: Theoretical and experimental study. Phys. Chem. Chem. Phys..

