# Supplementary material for: Halogen as Template to Modulate the Structures of the Nanocage-Based Silver(I)-Thiolate Coordination Polymers
Source: Molecules. 2026 Jan 19;31(2):331. doi: 10.3390/molecules31020331 (PMC12844137; doi:10.3390/molecules31020331)
Supplement: Supplementary file 1 [file molecules-31-00331-s001.zip › molecules-4025603-supplementary.pdf]

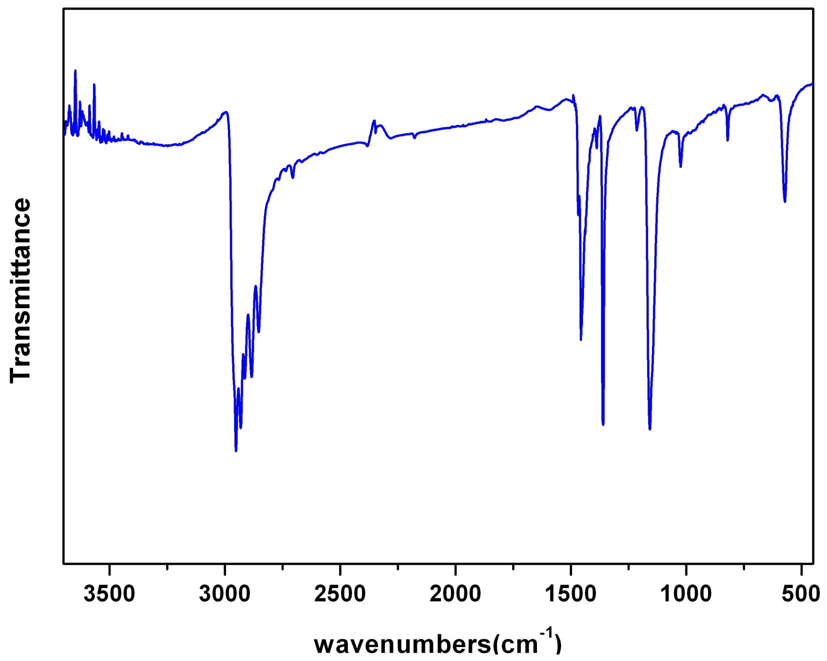

Fig. S1 FT-IR spectra of **USC-CP-2**.

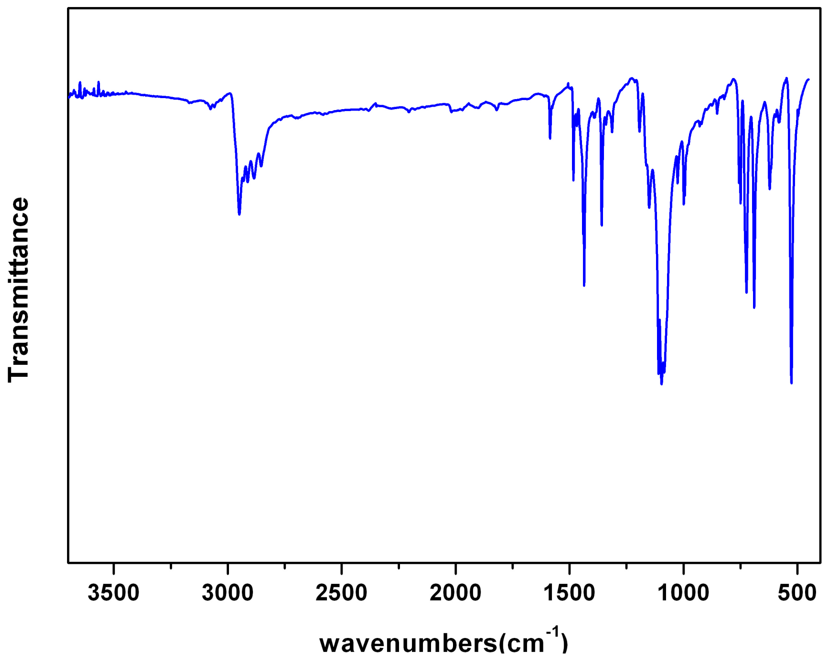

Fig. S2 FT-IR spectra of **USC-CP-4**.

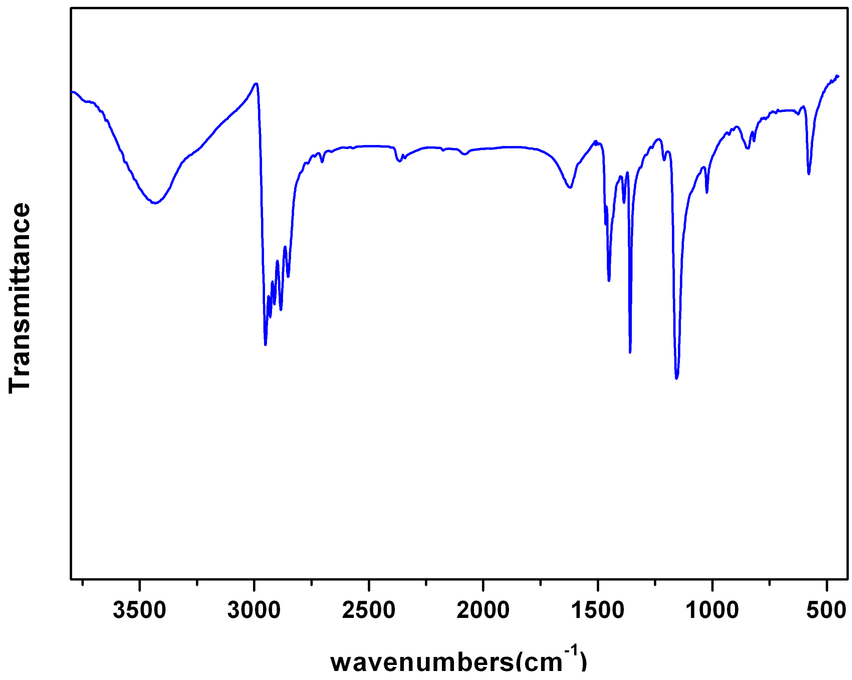

Fig. S3 FT-IR spectra of **USC-CP-3**.

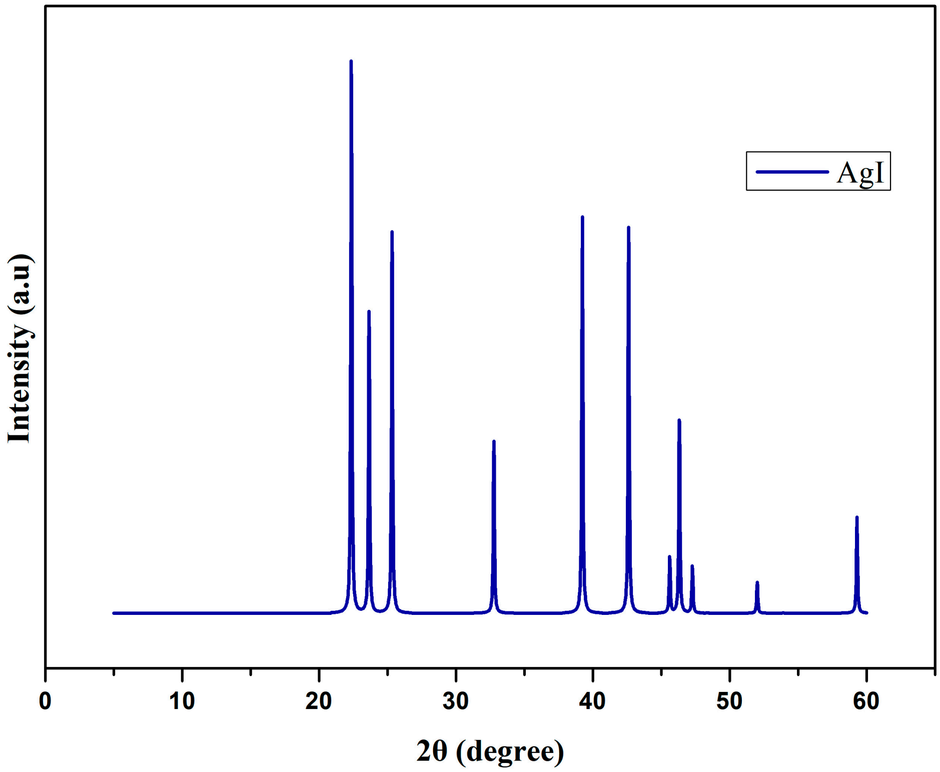

Fig. S4. Powder X-ray diffraction patterns of AgI.

Table S1. Bond lengths (Å) and angles(°) for **USC-CP-2.**

Bond	Dist.(Å)	Bond	Dist.(Å)
Ag(1)-S(1)	2.3685(13)	Ag(4)-Ag(5)	3.3355(7)
Ag(1)-S(3)	2.3774(13)	Ag(5)-S(5)	2.3705(13)
Ag(1)-Ag(5)	3.0585(6)	Ag(5)-S(6)	2.3786(13)
Ag(1)-Ag(3)	3.2510(7)	Ag(5)-Ag(6)	3.2087(7)
Ag(1)-Ag(4)	3.2744(6)	Ag(6)-S(4)#2	2.3851(12)
Ag(1)-Ag(2)	3.2965(7)	Ag(6)-S(6)	2.3874(13)
Ag(2)-S(2)	2.3997(13)	S(1)-C(1)	1.864(4)
Ag(2)-S(5)	2.3986(13)	S(2)-C(5)	1.851(3)
Ag(2)-Ag(3)	3.1139(7)	S(2)-Ag(3)#1	2.3943(13)
Ag(2)-Ag(5)	3.3033(7)	S(3)-C(9)	1.848(3)
Ag(3)-S(3)	2.3814(13)	S(4)-C(13)	1.864(3)
Ag(3)-S(2)#1	2.3943(12)	S(4)-Ag(6)#2	2.3851(12)
Ag(4)-S(4)	2.3895(12)	S(5)-C(17)	1.877(5)
Ag(4)-S(1)	2.3922(12)	S(6)-C(21)	1.856(3)
Ag(4)-Ag(6)	3.2054(7)		

Symmetry transformations used to generate equivalent atoms: #1 –x+1, -y+2, -z+1, #2 –x+1, -y+1, -z+2

Table S2. Bond lengths (Å) and angles(°) for **USC-CP-4**.

Bond	Dist.(Å)	Bond	Dist.(Å)
Ag(2)-S(2)	2.396(4)	Ag(3)-S(2)	2.378(5)
Ag(2)-S(1)	2.421(4)	Ag(3)-S(2)#4	2.378(5)
Ag(2)-Br(1)	2.871(2)	Ag(3)-Ag(1)#1	3.178(3)
Ag(2)-Ag(2)#1	3.220(3)	S(1)-C(1)	1.846(15)
Ag(4)-S(3)#2	2.371(8)	S(1)-Ag(4)#1	2.589(4)
Ag(4)-S(3)	2.389(8)	S(2)-C(5)	1.834(16)
Ag(4)-S(2)	2.576(4)	S(3)-S(3)#2	0.951(16)
Ag(4)-S(1)#1	2.589(4)	S(3)-C(9)	1.799(9)
Ag(1)-S(1)#3	2.386(4)	S(3)-C(11)#2	2.01(2)

Ag(1)-S(1)	2.386(4)	S(3)-Ag(4)#2	2.371(8)
Ag(1)-Ag(3)#1	3.178(3)	C(9)-S(3)#2	1.799(9)
Br(1)-Ag(2)#1	2.871(2)		

Symmetry transformations used to generate equivalent atoms: #1 –x+1/2, y, -z+7/4 #2 –x-1/2, y, -z+7/4 #3 –x+1, -y, z #4 –x, -y, z

Table S3. Bond lengths (Å) and angles(°) for **USC-CP-3**.

Bond	Dist.(Å)	Bond	Dist.(Å)
Ag(1)-S(6)#1	2.512(3)	Ag(5)-Ag(2)#2	3.4171(15)
Ag(1)-S(1)	2.527(3)	Ag(6)-S(4)	2.542(2)
Ag(1)-S(3)#2	2.669(2)	Ag(6)-S(5)	2.548(3)
Ag(1)-Ag(2)	3.0770(15)	Ag(6)-S(6)	2.551(3)
Ag(1)-I(1)	3.2099(13)	Ag(6)-I(1)	2.9914(12)
Ag(1)-Ag(7A)	3.239(9)	Ag(6)-Ag(7A)#1	3.310(7)
Ag(1)-Ag(4)#2	3.3442(15)	Ag(6)-Ag(7)#1	3.316(5)
Ag(2)-S(5)#1	2.488(3)	Ag(7)-S(5)	2.269(6)
Ag(2)-S(1)	2.519(3)	Ag(7)-S(6)#1	2.474(6)
Ag(2)-S(2)	2.695(2)	Ag(7)-Ag(7)#1	3.192(11)
Ag(2)-I(1)	3.2557(13)	Ag(7)-Ag(6)#1	3.316(5)
Ag(3)-S(2)	2.372(5)	Ag(7A)-S(6)#1	2.285(7)
Ag(3)-S(3)	2.388(5)	Ag(7A)-S(5)	2.502(7)
Ag(3)-Ag(5)#2	3.151(5)	Ag(7A)-Ag(7A)#1	3.161(14)
Ag(3)-Ag(4)	3.152(4)	Ag(7A)-Ag(6)#1	3.310(7)
Ag(3A)-S(3)	2.453(7)	S(1)-C(1)	1.835(12)
Ag(3A)-S(2)	2.484(7)	S(2)-C(5)	1.847(11)
Ag(3A)-Ag(4)	2.977(6)	S(2)-Ag(5)#2	2.401(2)
Ag(3A)-Ag(5)#2	2.980(7)	S(3)-C(9)	1.861(10)
Ag(3A)-I(1)#2	3.311(9)	S(3)-Ag(1)#2	2.669(2)
Ag(3A)-I(1)	3.320(8)	S(4)-C(13)	1.860(9)
Ag(4)-S(3)	2.404(2)	S(5)-C(17)	1.846(11)
Ag(4)-S(4)	2.405(2)	S(5)-Ag(2)#1	2.488(3)
Ag(4)-Ag(5)	2.9973(13)	S(6)-C(21)	1.846(11)

Ag(4)-Ag(1)#2	3.3442(15)	S(6)-Ag(7A)#1	2.285(7)
Ag(5)-S(2)#2	2.401(2)	S(6)-Ag(7)#1	2.474(6)
Ag(5)-S(4)	2.408(2)	S(6)-Ag(1)#1	2.512(3)
Ag(5)-Ag(3A)#2	2.980(7)	I(1)-Ag(3A)#2	3.311(9)
Ag(5)-Ag(3)#2	3.151(5)		

Symmetry transformations used to generate equivalent atoms: #1 –x, -y-2, -z, #2 –x, -y-1, -z
